# Optimizing Development of Plain Language Summaries of Clinical Trial Results

**DOI:** 10.3928/24748307-20260410-02

**Published:** 2026-07

**Authors:** Jennifer Doralt, Bernie Keohane, Adrienne McGilvray, Daniela Pobinger, Borislava G. Pavlova

**Affiliations:** a Baxalta Innovations GmbH, a Takeda Company, Vienna, Austria;; b Xogene Services LLC, Englewood, New Jersey;; c Takeda Development Center Americas, Inc., Cambridge, Massachusetts.

## Abstract

**Background:**

Plain language summaries (PLSs) play a crucial role in communicating complex clinical trial information to patients and the public. Due to the European Union Clinical Trials Regulation (EU CTR) 536/2014 requirement to disclose PLSs in parallel with the technical results, clinical trial sponsors will need to develop such summaries swiftly and efficiently, while avoiding potential obstacles. Their creation can be challenging for sponsor company stakeholders who typically use highly technical, scientific language.

**Brief Description of Activity:**

This manuscript describes the PLS development and review process established at our company. It also considers the collection of feedback via an anonymous survey, both of which were intended to optimize PLS preparation. Finally, it explores internal stakeholder preparedness for EU CTR requirements, and further opportunities to enhance PLS quality to better support patient centricity.

**Implementation:**

We observed a learning curve among stakeholders (content contributors and reviewers) and an enhanced appreciation for the PLS process.

**Results:**

This resulted in improved best practice principles for PLS writing, such as describing only the primary endpoint(s) and adverse drug reactions. The process of PLS development was streamlined following implementation of stakeholders' feedback received in PLS surveys circulated in 2023 and 2024. This led to increased satisfaction with the instructions provided for stakeholders and with the processes for PLS development, including the company-developed PLS template and glossary of plain language terms. Here we suggest that regularly adjusting PLS tools based on continuous feedback from internal stakeholders contributes to a more efficient and clear process.

**Lessons Learned:**

Optimization of the PLS development process using feedback from internal stakeholders supports efficiency, clarity, and patient-centricity, as well as ensuring preparedness for EU CTR requirements.

## Abstract (plain language summary)

**Plain Language Summary: **This article describes how the process for creating PLSs of clinical trial results has been improved, so they are clear, accurate, and consistent. Using standard formats, glossaries, and instructions, we worked to improve PLS quality using feedback from stakeholders. This approach promotes health literacy and public trust by making complex trial information accessible.

There has been a growing demand for greater transparency of information from clinical trials in language that can be readily understood by patients and the public (Barnes & Patrick, 2019; Getz & Farides-Mitchell, 2019; Kirkpatrick et al., 2017; Maurer et al., 2021). A survey showed that 91% of clinical study participants want to be informed of the trial results (Sood et al., 2009). Sharing results with trial participants and the broader community, using objective, non-promotional language aims to improve health literacy and build trust between the pharmaceutical industry, study participants, and the public (Getz & Farides-Mitchell, 2019; Gillow, 2015; Kurtz-Rossi et al., 2024; Lobban et al., 2022; Pal, Arnet, Elger, et al., 2024; Pal, Klingmann, Wangmo, et al., 2024; Wilcox et al., 2020). The value of plain language communication is increasingly recognized for its role in reducing barriers caused by limited health literacy (Stableford & Mettger, 2007). Limited health literacy has persisted as a policy-level challenge, having remained unaddressed for an extended period. Previously, the regulatory requirement was merely for sponsors to post information about clinical studies, including disclosing the results after study completion, on public registries. These were written in technical language, without consciousness of the health literacy levels of readers, and had regulatory compliance issues as well (Anderson et al., 2015; Goldacre et al., 2018).

However, the European Union Clinical Trials Regulation (EU CTR) 536/2014, drafted by the European Medicines Agency (EMA; 2014), now requires that lay summaries (plain language summaries, or PLSs) of clinical trial results (Annex V) be made publicly available via the Clinical Trials Information System (CTIS) portal. This requirement aims to address the longstanding lack of emphasis on inclusion and health literacy in information provided to the public. Provision of PLSs is compulsory for every interventional and lowinterventional clinical trial with at least one clinical site in the European Union or the European Economic Area (EU/EEA). Indeed, PLSs must be submitted to EMA simultaneously with the disclosure of the technical results on CTIS (EMA, 2014). Plain language summaries of study results registered on CTIS need to be uploaded within 12 months of study completion for adult trials and within 6 months for paediatric trials. This is a critical time constraint and increases the need for a streamlined PLS process. Additionally, while the EU CTR does not mandate translations, the European Commission Clinical Trials Expert Group (EC CTEG) recommends that the PLSs be provided in the official language of each of the countries where the trial took place (EC CTEG, 2021; EMA, 2014), adding another step to the process to reach a wider, more inclusive readership of PLSs.

Writing for the public is a challenge, as medical writers, clinical trial content contributors, and review teams in the pharmaceutical industry are accustomed to writing and reviewing technical documents (Edgell & Rosenberg, 2022). The EC CTEG and other initiatives provided guidance for writing PLSs of clinical trial results and the requirements are significantly different from the usual reporting standards (EC CTEG, 2021; European Commission, 2018; MRCT Center Post-Trial Responsibilities Workgroup, 2017). It is required that PLSs of clinical trial results provide a standalone, brief description of the clinical trial design, the primary objective(s), and the results in clear, easy-to-understand language so people with modest health literacy (a 6th to 8th grade reading level) can understand it the first time they read it (The MRCT Center of Brigham and Women's Hospital and Harvard, 2024).

Our aim was to establish a PLS development and review process that meaningfully engages our company stakeholders, advocating for its recognition as a ‘core competency’ to elevate PLSs' quality, advance health literacy, foster patient centricity, and strengthen our capacity to meet both regulatory requirements and the needs of the diverse and global patient populations we serve.

## Brief Description

Before the requirement for publication of PLSs became mandatory, we had developed an internal procedure that allowed PLSs to be used for company-related voluntary transparency efforts, while also ensuring future compliance with EU CTR and other regulatory requirements. This procedure involved internal stakeholders (e.g., sponsor company staff supporting the clinical trial in scope of PLS preparation) in the review process to refine PLSs' content and ensure accuracy, clarity, and patient-centric communication.

As this was a new type of document with specific requirements, the initial learning curve for clinical trial stakeholders was steep. Therefore, a new document preparation and review process was developed to make the process dynamic and to allow for continuous feedback and refinement from all stakeholders. By engaging internal stakeholders in the PLS preparation and evaluation process, we expected to enhance the effectiveness and impact of PLSs and ensure that PLSs meet internal requirements, reflect our organizational values, and resonate with target audiences (Zarshenas et al., 2023).

To collect feedback and evaluate preparedness of internal PLS stakeholders (content contributors and reviewers) we circulated anonymous surveys to them in 2023, and, following substantial process enhancements, conducted the survey again in 2024. Voluntary responses were requested concerning the preparation and review process, PLS content, and perceived utility of PLSs.

## Implementation

We began drafting PLS in 2014 as part of a pilot program. These initial PLSs were focused on health literacy, and a template was developed using input from patient advocacy programs and the PLSs were also tested using readability tests. In 2019, our Clinical Trial Transparency (CTT) team began development of PLS according to EU CTR 536/2014 requirements (EMA, 2014). As a first step, we established an internal process for development and review of PLSs, including, for a time, reviews by patient advocacy organizations. Based on review input from patients, and some internal content contributors such as patient-facing nurses, who could help identify the needs and interests of our patients, a tailored PLS template evolved that met these identified needs and interests and also complied with the 10 data elements from Annex V of the EU CTR 536/2014 regulation (The European Parliament and the Council of the European Union, 2014). We assembled PLS content contributors, reviewers and approvers, including clinical/medical/statistical/drug safety experts, intellectual property, CTT and regulatory experts.

The first step in PLS development was for CTT to prepare an initial PLS draft based on source documents such as clinical study reports (CSRs – sometimes in a draft state), statistical input such as Tables, Listings, and Figures (TLFs), statistical analysis plans (SAPs), informed consent forms (ICFs) and clinical protocols, as necessary.

Of the many components of a PLS that differ from standard clinical data reporting, the requirement to use easily understood medical terms (as opposed to the preferred terms from the Medical Dictionary for Regulatory Activities [MedDRA]) may present challenges, as the wording should be succinct, understandable to diverse and non-specialist audiences, and consistent. Existing publicly available plain language glossaries were found to be inconsistent, incomplete, or did not cover the therapeutic areas applicable to our company's products. Therefore, a glossary of plain language terms was developed, which has been referred to, expanded, and revamped since its inception in 2019. In this way, conversion of medical terms into easily understood language was standardized and made consistent.

Once an initial PLS draft was developed, it was circulated by CTT to stakeholders acting as content contributors and reviewers (among others: statistics and legal) via email. The email included instructions describing the rationale for developing PLSs, the required content (i.e., focus on primary endpoints and safety results, as per guidelines (EC CTEG, 2021), the scope of feedback requested from the stakeholders, and timelines for review. The email also noted that content-expert approval (clinical/medical and CTT) takes place before public disclosure on our clinical trial transparency web-site or any public registries.

The PLS development process and supporting tools (e.g., initial instructions, template and glossary) were assessed following feedback-driven activities to optimize the efficiency of PLS preparation and ensure preparedness for the EU CTR requirements (Pakarinen et al., 2022). To obtain empirical data on the PLS development process, an online survey tool was circulated twice, over 2 consecutive years. The scope of the survey was to collect stakeholders' feedback on the preparation and review process and evaluate the perceived clarity of PLS content. The questions in the survey related to the clarity of the plain language description of the study design, selected endpoints for reporting, and the presentation of the study results, with a special focus on the understandability of the safety results. Stakeholders were also asked about their perception of the utility of the PLSs in terms of understandability and usefulness to the target audience (study participants and the public). An open-ended question inviting survey takers to suggest interventions to help with the PLS review process was also included. All responses were anonymized. Stakeholder responses about the clarity of PLS principles and content were collected under three main categories: very clear, somewhat clear, and not so clear.

## Results

The PLS survey of stakeholders was circulated twice by CTT: in February/March 2023, and in March/April 2024. A total of 36 (in 2023) and 80 (in 2024) respondents anonymously provided information on the clarity of the PLSs' content and their feedback on the process.

The template, glossary, and initial instructions changed and were adapted based on PLS reviewer suggestions, particularly following the survey in 2023. Additionally, the use of source documents was revisited. As a result, while ICFs were utilized during the initial phase of PLS development, they were ultimately excluded as source documents, given that they are interpreted with the guidance of a health care professional (O'Sullivan L, 2020; Pietrzykowski & Smilowska, 2021).

The PLS template, created based on the EU CTR Annex V structure and content, was enhanced and amended after the 2023 survey to ensure simplicity and to better satisfy the needs of internal stakeholders and the audience. We introduced the “inverted pyramid” writing style to our template, which presented the conclusions and results of the trial first, followed by information on study design and methods once the lay reader's interest had been captured. The PLS template was further adjusted to display the original MedDRA terms in parentheses next to the plain language term from our glossary, so patients could refer to other publicly facing documents, such as those on ClinicalTrials.gov, and ensure data consistency. Company colors were removed from the template to prevent any content that could be construed as promotional, and complex graphics were excluded to allow broader applicability.

Concise plain language descriptions of company- and product-specific preferred terms were introduced to our internally developed plain language glossary, ensuring accuracy according to the MedDRA hierarchy but also consistency as per individual therapeutic area needs. The PLS glossary was enhanced after the survey in 2023 with additions of agreed-upon terminology of company-specific trial/product information for uniform use across relevant clinical trials.

**Figure 1.**
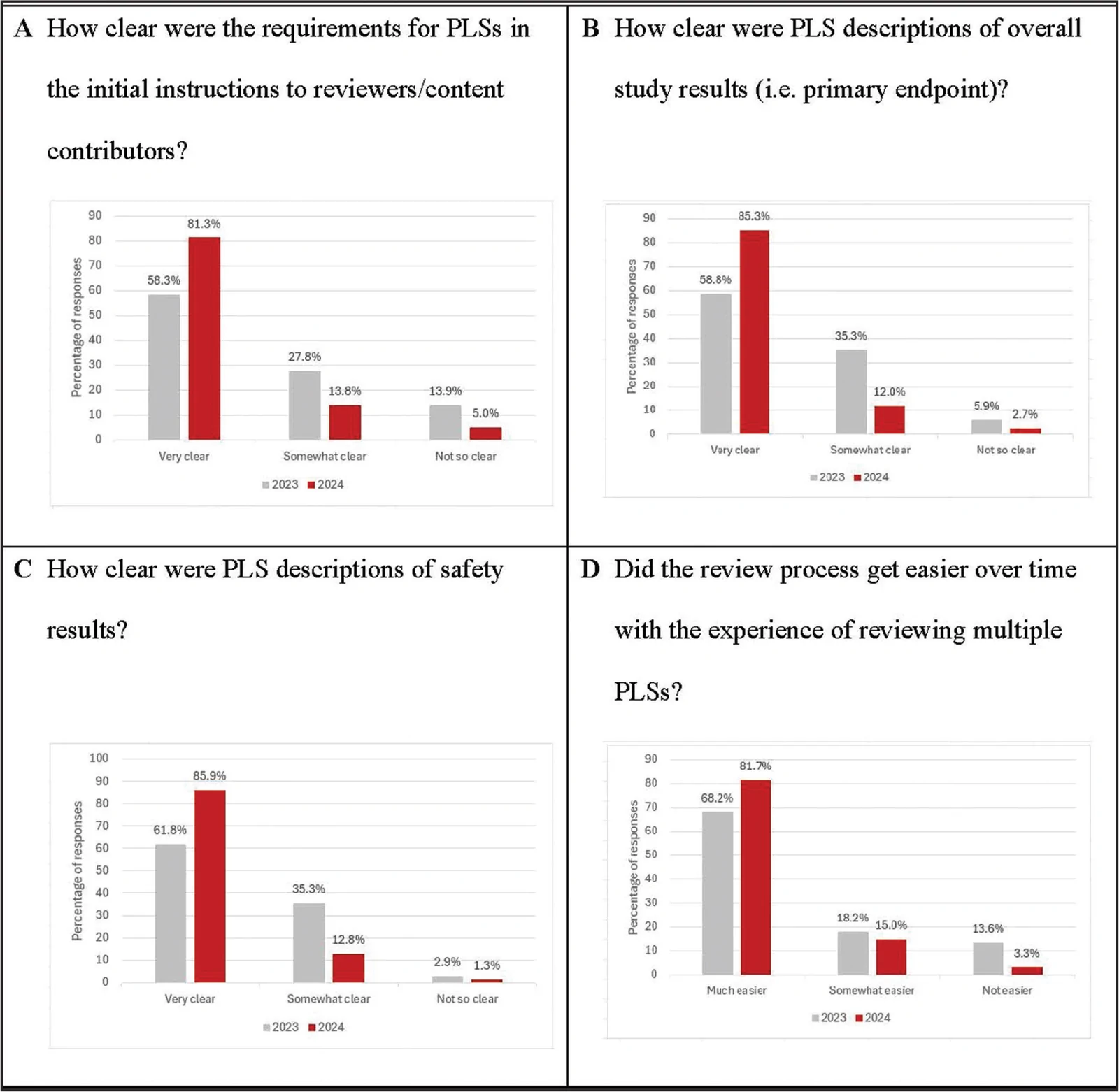
Survey results: assessment of the PLS development process over 2 consecutive years. PLS = plain language summary.

In addition, after the survey results from 2023, enhanced initial instructions (provided as an email at the initiation of PLS preparation) describing the rationale for developing PLSs, the required content as well as the stakeholder's role in the review were introduced, which had a positive impact on the survey results in 2024, as seen in **Figure 1A**. PLS stake-holders were reminded that the intended target audience for a PLS was a reader with no medical training, and/or a low level of health literacy. The review process was enriched with a set of criteria allowing for the identification and minimization of content issues, and the maintenance of health literacy principles and readability during PLS development. During management of PLS review after the 2023 survey, CTT received frequent feedback from content contributors requesting the presentation of Adverse Events (AEs) (referred to as “medical problems” in our PLSs) instead of Adverse Drug Reactions (ADRs/related adverse events), which are called “side effects.” Therefore, the review instructions and PLS template were adjusted to provide clear lay definitions of AEs versus ADRs and clarified that only ADRs/side effects were reported in the PLS as per EMA guidance. An exception to this rule is when AEs comprise a primary endpoint, in which case both ADRs (in the safety section of the PLS) and AEs (in the results section of the PLS) may be presented.

When asked how clearly PLSs described overall study results (i.e. the primary endpoint[s]), again, a trend indicating improvement was observed (**Figure 1B**). Comparable trends were seen with the responses regarding the clarity of the safety results presented in PLSs (**Figure 1C**). Stakeholders who had reviewed multiple PLSs were asked to provide their opinion on the ease of the review process over time or with experience (**Figure 1D**). Survey participants were more likely to find that the review process has gotten easier and more streamlined over time in 2024 compared to 2023.

Stakeholders were also surveyed regarding whether PLSs are a useful tool to increase clinical trial transparency for the public. These results were consistent between 2023 and 2024 (**Table 1**).

**Table 1:** Do You Think a Plain Language Summary Is a Useful Tool to Increase Clinical Trial Transparency for the Public?

**Perceived Usefulness Ratings**	**2023 (*N* = 36 respondents)**	**2024 (*N* = 80 respondents)**
	***n* (%)**	
Very useful	29 (80.6)	69 (86.3)
Somewhat useful	6 (16.7)	9 (11.3)
Not useful	1 (2.8)	2 (2.5)

Finally, respondents were given the opportunity to write suggestions for improvement at the end of the survey (as an open question in a free text field). Respondents to the first survey in 2023 requested that PLS development be considered a milestone among the overall post-study completion activities and timelines to allow for sufficient review time, which was later integrated into our PLS process. Suggestions - including conducting PLS reviews within a document management system (to enable a collated response rather than individual email feedback), better planning of PLS development at the onset of CSR drafting (to facilitate submissions in parallel with technical trial results), and clarifying in the PLS that patients should not make treatment or study participation decisions based on a single clinical trial and should consult their doctors before changing treatments - were integrated into the overall strategy.

## Lessons Learned, Limitations and Implications

This report discusses our preparation for and experience with PLS development and implementation process in anticipation of the regulatory requirement to publish PLSs for all studies under EU CTR, as well as for public disclosure on an externally facing company-owned website. A key component of our preparation was the survey to determine stakeholders' preferences and to collect input on our process and PLS preparation tools. The survey sought to engage stakeholders to improve the development process, without compromising objective clinical trial information in the PLS that is understandable and meaningful to a non-medical audience. In addition to PLS process improvement and clarity, we used the survey results to enhance our PLS template, update company and therapeutic area specific terminology in our glossary of plain language terms, and optimize instructions provided for initial reviews of PLSs.

To overcome the barriers associated with limited health literacy, the effective and adequate communication of clinical trial results must be prioritized by sponsor companies and the main stakeholders involved, given its critical impact on patients' ability to make informed health decisions. Part of the rationale for engaging stakeholders in the development process was to ensure they acquired the necessary knowledge to effectively review PLSs, comprehend the intended objectives, and foster a shared understanding of the needs of the PLSs' target audience. The PLS stakeholders expressed satisfaction with the clarity of the goals in developing PLSs according to the original process, while the clarity regarding the needs of the intended audience improved after some process enhancements as shown in the survey results from 2024.

The EU CTR Annex V originally proposed presenting only the primary endpoints pre-specified in the study's statistical design and ADRs (European Commission, 2018). However, we decided to follow current trends that favor presenting primary endpoints and ADRs but also results that could be of particular interest to the patient population, such as other safety endpoints, patient-reported outcomes, or in exceptional cases, efficacy endpoint(s), with a rationale. We enhanced the PLS template to ensure that endpoint presentation was compliant with regulations, facilitated patient accessibility to the most relevant trial information, and stipulated when to mention secondary endpoints—e.g., in vaccine studies, when the primary endpoint is safety, but the reader is interested in whether it produced the desired immune response, that the secondary endpoint be mentioned as “additional” information. At the same time, we clarified the PLS template text with user instructions (which clearly improved the understanding of the requirements to consider when reviewing PLSs) and color-coded sections, delineating standard template text and citing specific tables in the CSR as sources, to ease review.

We also tried to encourage reviewers to see the PLS from a trial participant's viewpoint. After all, basic, objective information of interest to patients concerning safety and effectiveness enables the reader to make informed decisions about future treatment options and has implications concerning informed consent for future trials. Indeed, PLSs may affect the informed consent process for clinical trial participation if patients are influenced by incomplete or decontextualized explanations—particularly when trial results are inconclusive. If patients overestimate the reliability or validity of information presented in a phase 2 trial PLS, for example, they may make a misinformed choice to enroll in a subsequent phase 3 trial. While necessarily brief, plain language protocol synopses that are publicly available on CTIS should, in addition to PLS results, also help patients make informed decisions as to whether to participate in clinical trials. Indeed, observational studies examining how PLSs influence the informed consent process, including patients' interest in, and perceived voluntariness of, enrolling in follow-up studies after reading results from a precursor trial, could provide valuable insights.

According to PLS readers, key messages in a PLS should include (a) whether the intervention was effective and (b) what side effects were experienced. Indeed, the PLS audience may have different needs than what sponsors assume, as is apparent when patients have a say in their health care treatments (Say & Thomson, 2003). The “inverted pyramid” writing style, which we introduced post 2023 survey results to our template, may have contributed to the substantial increase in satisfaction of PLS stakeholders in 2024. The number of survey respondents who found that the PLS review process became easier and more streamlined over time increased from 68.2% in 2023 to 81.7% in 2024 (**Figure 1D**). This approach was perceived by stakeholders as ensuring that even readers who only read the first PLS paragraph still understood the main outcome of the trial.

In addition, a consistent approach in the use of medical language and terms is now more important than ever, as regulatory documents such as clinical trial protocols and CSRs have become available in the public domain because of increasing transparency requirements. Therefore, the PLS reader should be able to refer to various publicly available clinical trial documents with some understanding of medical terminology and see familiar terms there (i.e. plain language terms next to technical terms in parentheses). We considered it “best practice” to provide the original medical/technical term in parentheses, for clarity, which was implemented in the PLS template after the 2023 survey and was reflected by reviewers'/content contributors' satisfaction regarding usefulness of PLSs to increase clinical trial transparency for the public in the 2024 survey.

To retain consistency between our PLSs and these other sources available in the public domain, we recommend creation of a company-owned plain language “glossary” of scientific terms in simple, easy to understand language, which can be expanded upon and referred to by the PLS stakeholders. The implementation of such a glossary after the 2023 survey streamlined the PLS review process, helping to reduce or avoid time-consuming discussions about changes or inconsistencies across PLSs in a specific therapeutic area. This has supported consistency but also eased the burden on PLS writers and stakeholders, giving them a source for frequently used company/product-specific complex terms. This approach contributed to the improved 2024 survey results on the understandability of trial safety in the PLSs (**Figure 1C**).

Also, to allow for broader utility of PLSs, complex graphics were not considered necessary in our current template. The United Kingdom Health Research Authority (HRA) requires PLSs to be embedded in the Clinical Study Report regulatory submission form, without complex figures or graphics (HRA, 2025). Patient advocacy organizations reviewing our template also suggested that such graphics may not be considered best practice in PLSs (Dormer et al., 2022).

Our template also had a field where URLs were included for direct access to additional clinical trial related information, such as public disclosure databases (e.g., ClinicalTrials. gov) and relevant scientific publications. Currently, we share PLSs with patients and the public via our externally facing website, where CSR synopses, links to trial summary results on public registries and other specific information in plain language are also available to broaden access to clinical trial resources.

Although the survey was useful in helping us improve PLSs, its chief limitation was the lower number of survey responders in 2023 (*N* = 36) compared to those in 2024 (*N* = 80). While participation in the survey was voluntary and the appeal for engagement was sent to mostly the same PLS content contributors and reviewers, we observed an increased response rate following the enhancement of tools and processes after the 2023 survey. This observation could be interpreted as having been driven by increased reviewer motivation to provide a perspective on process and tool improvements upon seeing the results of critical reviews captured following the first survey. Furthermore, by 2024, the EU CTR requirements had become a reality, with new clinical trials and nearly all ongoing trials in the EU/EEA being initiated or transitioned under the CTIS process, highlighting the critical need for fast and efficient PLS preparation to stay in compliance with the publication requirements.

A limiting factor in our PLS preparation process is the lack of involvement of the patient audience in the review process. During the early stages of process development, we collaborated with patient advocacy groups to review every PLS. However, the current regulatory landscape poses challenges, particularly with tight timelines, making it difficult to effectively incorporate external input.

Stakeholders responding to our survey suggested in the free text field that PLS development should be included among the overall post-study completion activities and time-lines so that sufficient reviewer resources could be planned. This is a significant point, especially as the same functional reviewers and PLS content contributors are involved in many parallel activities at or post-study completion (e.g., during final TLFs and CSR preparation). As the clinical trial technical results disclosure and the PLS posting on CTIS will be required simultaneously, PLSs will be developed in parallel to the early stages of CSR preparation. While concerns were expressed that sufficient time should be allowed for dialogue back and forth during PLS development, there was usually not enough time to review several drafts. This was only possible when the process started with draft TLFs. Based on survey feedback regarding timelines, a PLS shell (or “mock report”) was developed from the trial protocol or CSR shell (“mock CSR”). Specific trial data was added to the PLS shell later, upon TLFs finalization. The PLS was then updated with results from the mature CSR and cross-checked with the final CSR. This approach allowed for simultaneous review and approval of the PLS and CSR, reducing the time constraints associated with submission requirements.

Indeed, as the PLS stakeholders also reviewed the results that were to be posted on public registries (e.g., EudraCT/ CTIS and ClinicalTrials.gov), the timelines for PLS review were built in tandem with the review of the technical results. Additional time was allocated for translations, as the EC CTEG stipulates that PLSs be translated into all official country languages in which the study was conducted (EMA, 2014).

However, the simultaneous work on CSR, technical trial results, and PLSs while maintaining quality and efficiency requires massive resource and time investments from study teams. This drives the need to explore further ways for process optimization and increased productivity to improve the experience of PLS content provision as well as the quality of the final PLS. New AI technology could further ease development of PLSs and improve PLS quality (Edwards, 2025; Van Veen et al., 2024).

## Conclusions

Our survey results indicated that measures should be taken at each step of the PLS development process, but particularly at the outset, to ensure effective communication with the internal stakeholders and ensure that they become accustomed to the process of writing PLSs. Survey results demonstrated that reviewers/content contributors deemed the intended reader and the overall aims of plain language summaries important and found these areas to have improved over time in our PLSs. The objective was not merely the simplification of complex scientific terminology, but the elucidation of scientific and medical details while providing context. We advocate that institutions engage in communication and idea-sharing in this emerging field to enhance the PLS structure and align content with health literacy principles to better inform about potential risks, support participants' consent and decision-making, and present a clearer patient-relevant picture, while establishing industry best practices and maintaining the highest standards of transparency. By developing tailored standard PLS templates, maintaining company-owned glossaries for consistency, and gathering feedback from internal stakeholders, sponsors can enhance the efficiency and the overall quality of PLSs, ensuring that they remain patient-centered and prepared for the EU CTR requirements.
